# Supplementary material for: Bacteriophage T5 tail tube structure suggests a trigger mechanism for Siphoviridae DNA ejection
Source: Nat Commun. 2017 Dec 5;8:1953. doi: 10.1038/s41467-017-02049-3 (PMC5717097; doi:10.1038/s41467-017-02049-3)
Supplement: Supplementary file 1 — Supplementary Information [file 41467_2017_2049_MOESM1_ESM.pdf]

a

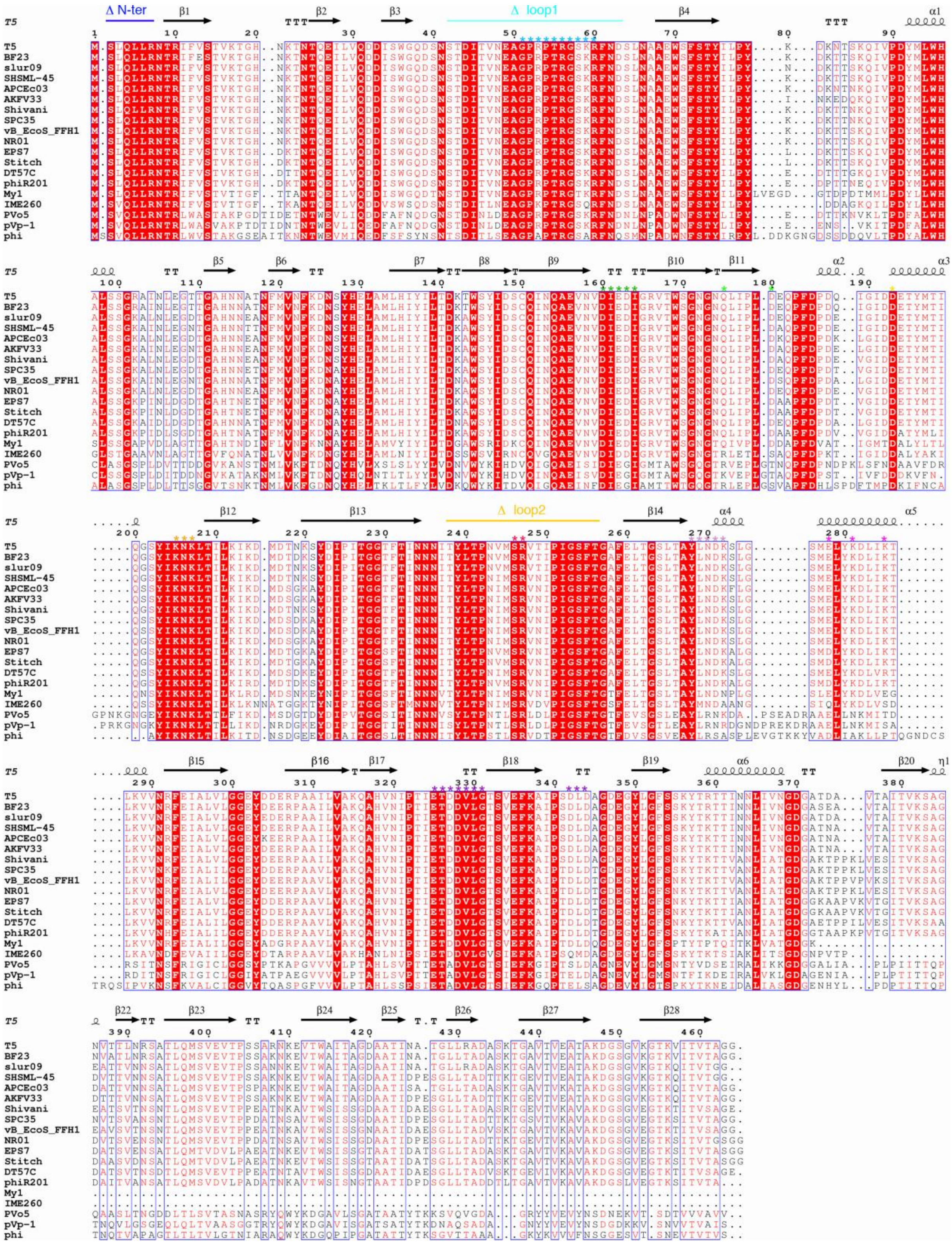

b

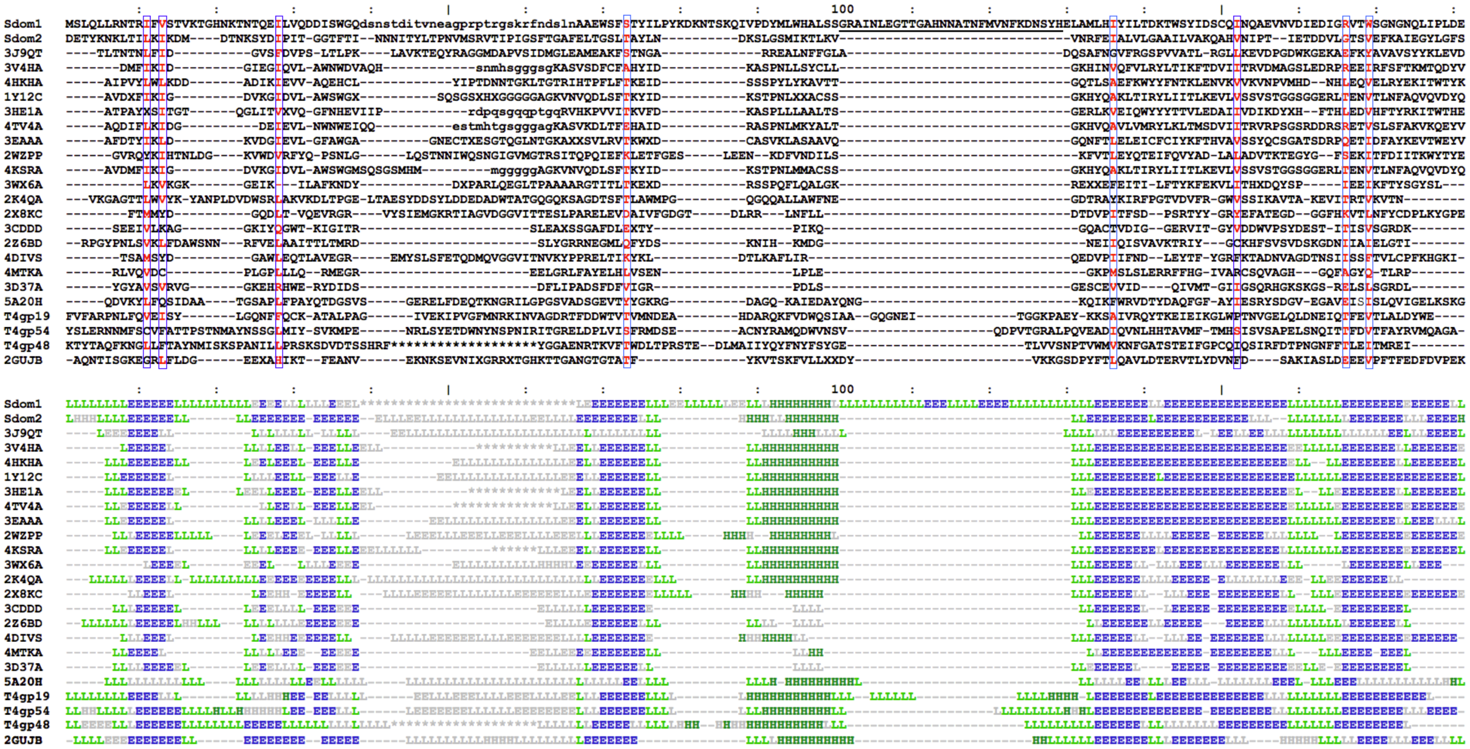

Supplementary Figure 1: **a. Amino acid sequence alignment of TTPs from T5-like bacteriophages**, displayed using EsPript[2] (%Equivalent option). The amino acids mutated to alanines in the different pb6 mutants are marked by asterisks with the same colour code as in Figure 4 and Supplementary Tables S4 and S5. **b. Pairwise DALI[3] sequence alignments of phage, T6SS and pyocin proteins on subdomain1** (Sdom1). **The upper panel** shows the amino acid sequence alignments, presented using EsPript[2] and combining the Risler, % Multalin and % Equivalent options (threshold = 0.7). All other options did not highlight any residue in the alignment. In lowercase are indicated the sequences of the long loops that are not ordered. In T4gp48, asterisks stand for the 55 aminoacid unstructured loop. The underlined insertion in subdomain 1 is the coloured magenta in Figure 1a. **The lower panel** presents the secondary structure assignments by DSSP (H: helix (green), E: strand (blue), L: coil, *: undefined, coloured when common). For clarity, insertions in the structural homologues of pb6 were not expanded. Sdom2: pb6 subdomain2; 3J9Q: inner tail tube protein of *Pseudomonas aeruginosa* pyocin; 3V4H: inner tube protein (Hcp) of the T6SS of *Yersinia pestis*; 4HKH: inner tube protein of the T6SS of *Escherichia coli*; 1Y12: inner tube protein of the T6SS of *Pseudomonas aeruginosa*; 4TV4: inner tube protein of the T6SS of *Burkholderia pseudomallei*; 3EAA: inner tube protein of the T6SS of *Edwardsiella tarda*; 2WZP: Distal tail protein of siphophage p2; 4KSR: inner tube protein of the T6SS of *Vibrio cholerae*; 3WX6: inner tube protein of the T6SS cluster 1 of *Burkholderia pseudomallei*; 2K4Q: tail tube protein of siphophage λ; 2X8K: Distal tail protein of siphophage SPP1; 3CDD: baseplate hub protein (subdomain 1) of promyophage MuSO2 from *Shewanella oneidensis*; 2Z6B: baseplate hub protein (gp27, subdomain 1) of myophage T4; 4DIV: Distal tail protein of siphophage TP901.1; 4MTK: central spike (VgrG1, subdomain 1) of the T6SS of *Pseudomonas aeruginosa*; 3D37: tail tube protein of a pro-siphophage of *Neisseria meningitides*; 5A20: tail tube protein of siphophage SPP1; T4gp19: inner tail tube protein of myophage T4; T4gp54 and 48: inner tail baseplate proteins of myophage T4; 2GUJ: tail tube protein of prophage PBSX of *Bacillus subtilis*.

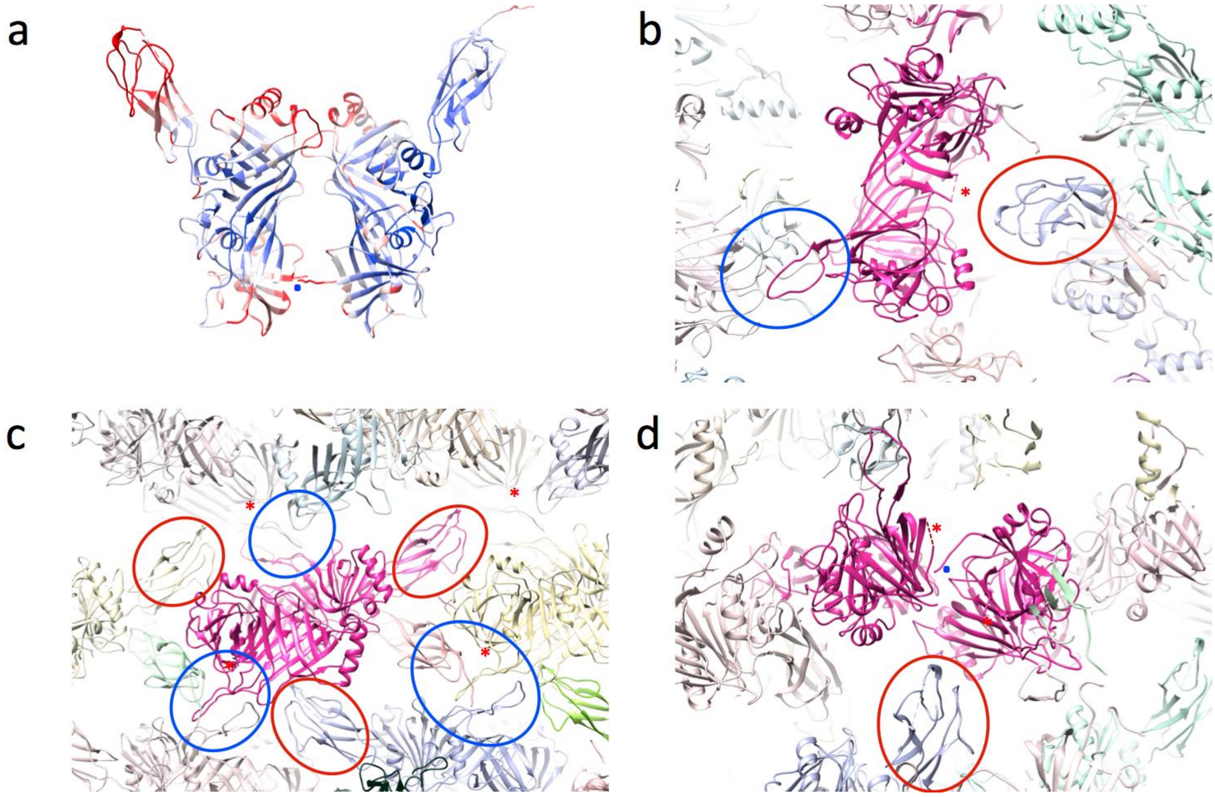

Supplementary Figure 2: **pb6 crystal packing. a.** Ribbon representation, coloured according to the average residue temperature factor (red: more flexible, blue: more rigid) of the asymmetric unit of the pb6 crystal. Both monomers are superimposable with an rmsd of 0.506 Å over 376 residues. This value drops down to 0.355 Å over 332 residues when the Ig-like domain and its linker are omitted. In one monomer, the Ig-like domain appears very flexible and less resolved (high temperature factor, left). This is due to it pointing towards a solvent channel in the crystal (circled red in b-d). In the other monomer, the Ig-like domain is much better resolved (lower temperature factor, right), as it takes part, also through the C-terminal His tag, in crystal contacts with the long loop β13-β14 (circled blue in b-c). **b-d.** Crystal packing of pb6. The long loop of subdomain 2 (β13-β14) of both monomers form crystal contacts, together with the Ig-domain (circled blue), thus constraining it. The long β3-β4 loop of subdomain 1 of both monomers points towards the solvent and is not ordered (indicated by a red asterisk in b-d), as is also observed in solution for the TTP of phage λ[4]. In gpV, the N-terminus is also disordered; in the pb6 crystal however, the N-terminus of one of the monomers also takes part in crystal contacts (blue dot in a and d) and is thus resolved. It still appears flexible, however, as shown by its high temperature factor (blue dot in a).

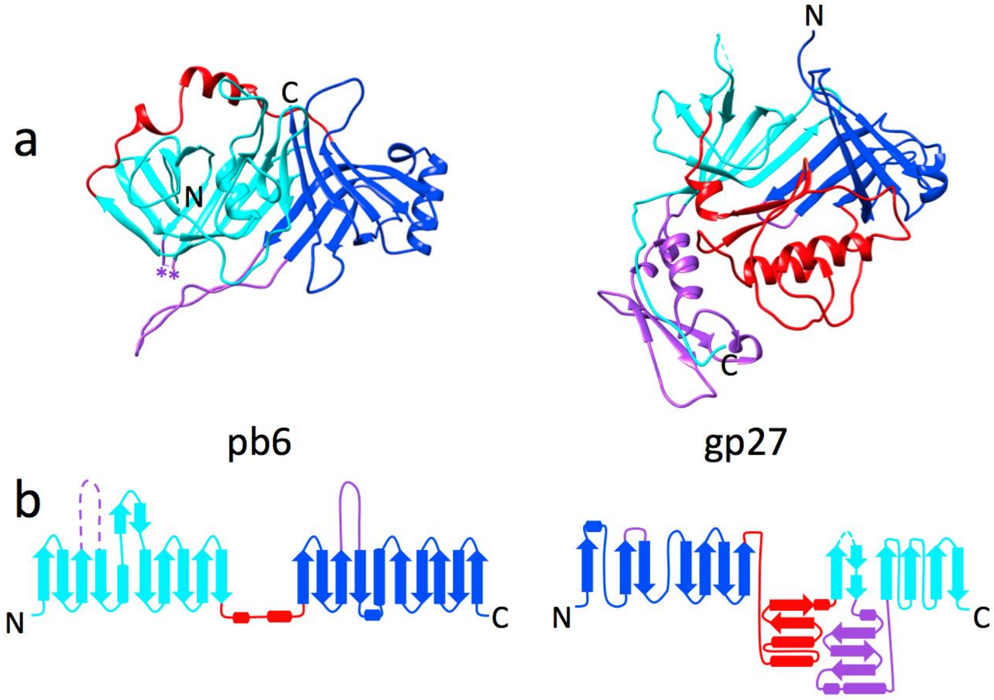

Supplementary Figure 3: **Independent domain duplication in pb6 and gp27. a.** Ribbon representation of pb6 (left) and T4 gp27 (right, PBD 2Z6B), in the same side view orientation. T4 gp27 was chosen as a representative of Myophage hub and T6SS spike proteins. In the latter, the C-terminus is fused to a spike domain. In pb6, the Ig-like domain and its linker have been removed for clarity. pb6 subdomains 1 and 2 and their linker are coloured as in Fig 1 (cyan, blue and red, respectively), and the long loops of the two subdomains are coloured purple; the start of the unstructured subdomain 1 long loop is indicated by asterisks. The same colour code was used for structurally homologous features in gp27. **b.** Topology diagram of pb6 (left) and gp27 (right). The equivalent of the pb6 long loop in gp27 subdomain 2 is short; in the other subdomain it is much longer and forms, together with the linker between the two subdomains, another domain that serves the function of the protein[5]. Furthermore, the link between the two subdomains is topologically different in the two proteins, as it connects the C-terminus of subdomain 1 to the N-terminus of subdomain 2 in pb6, and the N-terminus of subdomain1 to the C-terminus of subdomain2 in gp27. N and C: N- and C-terminus respectively.

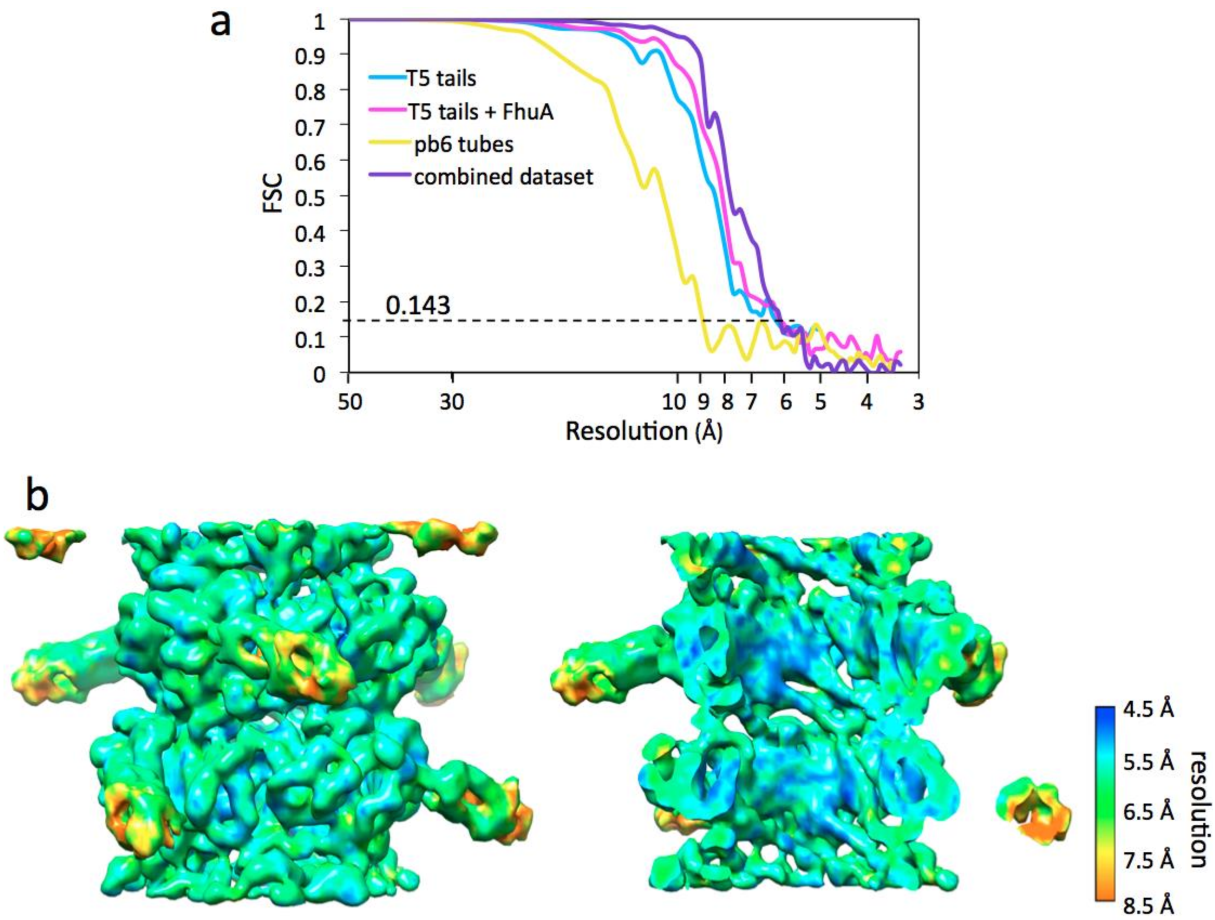

Supplementary Figure 4: **EM data. a.** Fourier shell correlation curves for reconstruction of the full (cyan) and empty (magenta) tails, of the combined dataset (purple) and of pb6 tubes (yellow). **b.** Local resolution of the combined density map shown on the exterior side view of the tube (left) or on its interior (right).

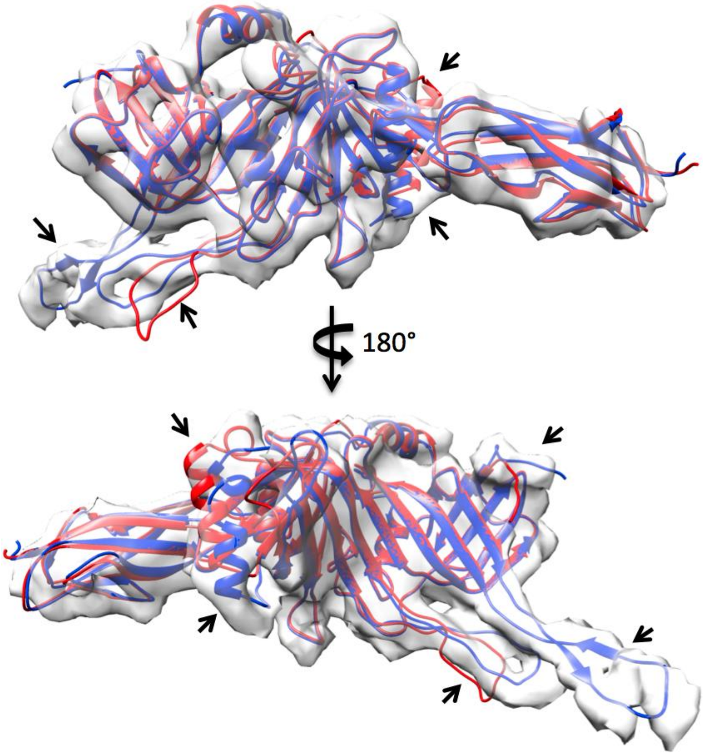

Supplementary Figure 5: Flexible fitting of pb6 crystals in a segment of the EM map of the tail. Ribbon representation of the crystal structure of the pb6 monomer rigidly (red) and flexibly (blue) fitted into a segment of the combined EM tail tube density. Main differences occur in the N-terminus, the 2 large loops and the α5 helix (black arrows).

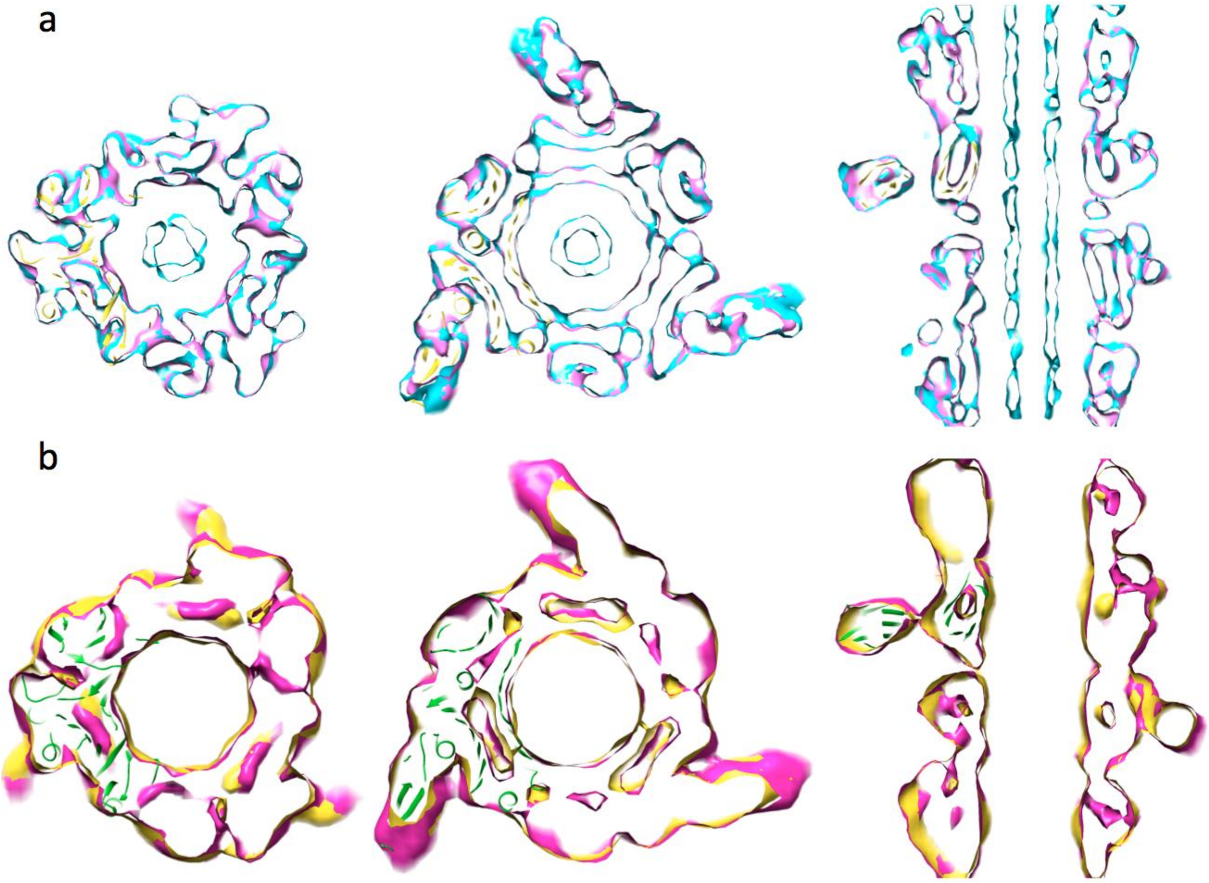

Supplementary Figure 6: **Comparison of full tails, empty tails and pb6 fibres.** Three sections through the overlaid EM densities to allow comparison of the full (cyan) and empty (magenta) tail tube at 6 Å resolution (**a)**, and the empty tail tube (magenta) and the tube fibres (yellow) at 9 Å resolution (**b**). Depicted as a yellow (a) and green (b) ribbon is the crystal structure of pb6 rigidly fitted into the density of the tubes. See also Supplementary Movies S1 and S2.

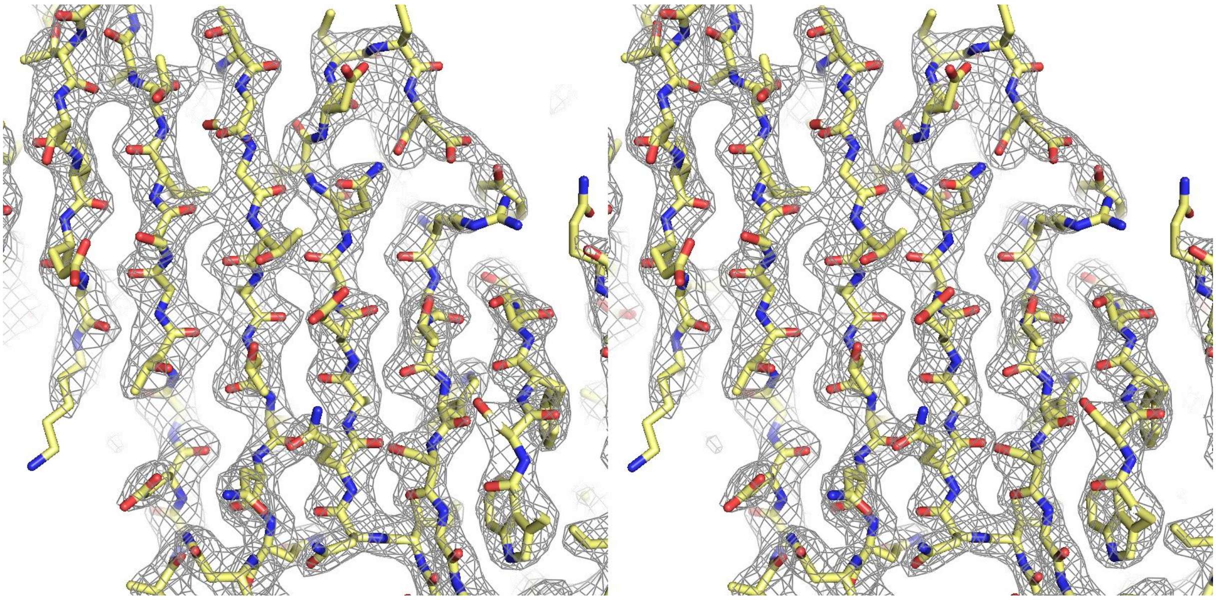

Supplementary Figure 7: **Stereo image of a portion of the 2Fo - Fc electron density map** displayed as a map with a contour of 1.5 σ**.**

Supplementary Table 1: Result of the DALI query on pb6 subdomains 1 (residue 1-192) and 2 (residue 193-374).

	Subdomain 1				Subdomain 2				
PBD	Z-score	rmsd	lali	% id	Z-score	rmsd	lali	% id	Protein
	6.1	3.3	98	7					pb6 subdomain 2
3J9Q	7.5	2.6	97	10	5.6	3.8	111	8	*Pseudomonas aeruginosa* Pyocin inner TTP
3V4H	7.3	2.8	95	13	7.2	3.0	102	9	*Yersinia pestis* T6SS Hcp1
4HKH	7.2	2.8	96	7	7.8	3.6	115	9	*Escherichia coli* T6SS Hcp1
3HE1	7.1	3.1	98	10	8.4	3.4	108	10	*Pseudomonas aeruginosa* T6SS Hcp3
2WZP	7.1	3.3	99	10	6.1	4.4	120	11	Siphophage p2 distal tail protein
1Y12	6.8	2.9	97	18	7.0	3.1	113	13	*Pseudomonas aeruginosa* T6SS Hcp1
4TV4	6.6	3.0	94	13	7.3	3.4	103	11	*Burkholderia pseudomallei* T6SS Hcp1
3EAA	6.5	2.9	97	10	7.1	3.4	117	14	*Edwardsiella tarda* T6SS Hcp1
4KSR	6.5	2.9	97	16	6.9	2.8	108	13	*Vibrio cholerae* T6SS Hcp1
3WX6	6.3	2.3	83	6	6.0	2.8	89	15	*Burkholderia pseudomallei* T6SS Hcp1
2X8K	5.8	3.6	88	13	5.3	3.9	100	14	Siphophage SPP1 distal tail protein
3CDD	5.7	2.8	76	7	5.0	4.2	89	13	*Shewanella oneidensis* promyophage MuSO2 baseplate hub protein
2Z6B	5.4	3.5	90	7	4.2	3.7	98	12	Myophage T4 baseplate hub protein (gp27)
4MTK	5.3	2.6	76	4	5.4	3.4	95	9	*Pseudomonas aeruginosa* T6SS central spike (VgrG1)
2K4Q	5.1	4.0	100	12	5.1	4.9	101	10	Siphophage λ TTP
1WRU	5.0	3.5	83	12	3.7	4.1	91	12	Myophage Mu baseplate hub protein
2GUJ	5.0	3.1	87	1	5.9	3.9	100	14	*Bacillus subtilis* prophage PBSX TTP
4DIV	4.5	3.0	86	5	3.5	4.2	94	9	Siphophage TP901.1 distal tail protein
3D37	4.1	3.8	84	8					*Neisseria meningitides* prosiphophage TTP
5A20	3.6	3.0	92	5	5.0	4.0	107	11	Siphophage SPP1 TTP
5IV5 T4gp19	7.8	4.3	112	5	4.9	4.1	114	5	Myophage T4 inner TTP
5IV5 T4gp48	5.7	3.5	101	5	6.2	3.5	111	10	Myophage T4 baseplate TTP
5IV5 T4gp54	7.4	3.2	109	6	5.9	3.9	119	13	Myophage T4 baseplate TTP

rmsd: root-mean-square deviation of Cα atoms in the least-squares superimposition of the structurally equivalent Cα atoms, lali: number of structurally equivalent residues, % id: percentage of identical amino acids over all structurally equivalent residues.

Supplementary Table 2: Cryo-EM data collection and image analysis statistics

**Sample**	**Full tails** (EMD-3689)	**Empty tails pb6** (EMD-3689)	**pb6 tubes** (EMD-3691)
Magnification	15,500	23,000	23,000
Voltage (kV)	300	300	300
Defocus range (μm)	-1.0 / -7.0	-1.5 / -4.0	-1.5 / -5.0
Number of micrographs	61	84	53
Pixel size (unbinned) (Å)	1.24	0.82	0.82
Number of frames per movie	40	40	40
Total dose per movie (e^-/$Å^2$)	40	40	40
Frame alignment	Digital Micrograph	MotionCorr	Digital Micrograph
Symmetry imposed	3-fold	3-fold	3-fold
Number of helical segments picked	32891	50014	19392
Number of segments in final reconstruction	24243	22704	12244
Resolution (Å) FSC = 0.143	6.2	5.8	8.8

Supplementary Table 3: Summary of the performed mutants.

Name	Mutation (protein)	Tube formation	Fraction of soluble protein (%)
pb6-full length		+	21 ± 4 (n = 5)
pb6-Ig	p.S2_D374*del*	nr	nr
pb6-ΔIg	p.A375_464*del*	+	18
pb6-ΔNter	p.S2_N8*del*	-	96
pb6-Δloop1	p.S42_D63*del*	-	92
pb6-Loop1A	p.G51A_P52A_R53A_P54A_T55A_R56A_G57A_S58A_K59A_R60A	ni	97
pb6-160A	p.D160A_I161A_E162A_D163A_I164A	ni	91
pb6-QD	p.Q175A_D180A	+	24
pb6-D	p.D193A	+	26
pb6-Δloop2	p.T238_A256*del*	-	91
pb6-KNK	p.K205A_N206A_K207A	+	69
pb6-SR	p.S246A_R247A	+	74
pb6-270A	p.Y268A_L269A_N270A_D271A_K272A	ni	82
pb6-E	p.E278A	+	47
pb6-K1	p.K281A	+	15
pb6-K2	p.K285A	+	48
pb6-3K	p.E278A_K281A_K285A_K288A	+	60
pb6-330A	p.E325A_T326A_D327A_D328A_V329A_L330A_G331A_D342A_L343A_D344A	ni	74

p. indicates point protein mutations. X_Ydel indicates an X-Y deletion, nr: not relevant, ni: not investigated. +: tube formation and -: no tube formation, as determined by negative stain EM of the insoluble fraction after cell lysis, in buffer conditions that favour tube formation. The fraction of soluble protein was determined by comparing the amount of soluble and insoluble pb6 in bacterial lysate on SDS-PAGE (Fig. 4b) using the gel analysis tool in ImageJ[1]. Mutants are colour coded as in Figure 4 and Supplementary Figure 1a.

Supplementary Table 4: Primers used for generating the pb6 mutants.

Mutant	Primer1	Primer2
pb6-Ig	CTTTAAGAAGGAGATATACATATGGC TGTAACCGCAATTACTG	CAGTAATTGCGGTTACAGCCATATGTATA TCTCCTTCTTAAAG
pb6-ΔIg	GGTGACGGTGCTACTGATGCTGGCGC TGGCCATCATCATCATCATCATTGAG CTGTAACCGCAATTACTG	CAGTAATTGCGGTTACAGCTCAATGATGA TGATGATGATGGCCAGCGCCAGCATCAG TAGCACCGTCACC
pb6-ΔNter	CTTTAAGAAGGAGATATACATATGAC TCGAATCTTCGTGTCTACG	CGTAGACACGAAGATTCGAGTCATATGT ATATCTCCTTCTTAAAG
pb6-Δloop1	CTTGGGGTCAGGACAGTAACTCTTTA AACGCAGCTGAGTGG	CCACTCAGCTGCGTTTAAAGAGTTACTGT CCTGACCCCAAG
pb6-Loop1A	CAGATATTACTGTTAATGAAGCTGCG GCGGCGGCGGCGGCGGCGGCGGCGG CGTTTAACGACTCTTTAAACGCAG	CTGCGTTTAAAGAGTCGTTAAACGCCGCC GCCGCCGCCGCCGCCGCCGCCGCAGCTTC ATTAACAGTAATATCTG
pb6-160A	GATTAATCAGGCAGAAGTTAACGTTG CGGCGGCGGCGGCGGGTCGTGTAAC TTGGTCTG	CCAGACCAAGTTACACGACCCGCCGCCG CCGCCGCAACGTTAACTTCTGCCTGATTA ATC
pb6-QD	CTTGGTCTGGTAATGGTAACGCGCTA ATCCCACTGGCGGAGCAACCATTTGA TCCAG	CTGGATCAAATGGTTGCTCCGCCAGTGGG ATTAGCGCGTTACCATTACCAGACCAAG
pb6-D	CCAGATCAAATAGGTATTGATGCGGA AACTTATATGACTATCCAG	CTGGATAGTCATATAAGTTTCCGCATCAA TACCTATTTGATCTGG
pb6-Δloop2	CTTTTACTATTAACAACAACATTGGT GCTTTTGAATTAACTGG	CCAGTTAATTCAAAAGCACCAATGTTGTT GTTAATAGTAA
pb6-KNK	CTATCCAGGGTTCTTACATTGCGGCG GCGCTAACGATCTTGAAGATCAAG	CTTGATCTTCAAGATCGTTAGCGCCGCCG CAATGTAAGAACCCTGGATAG
pb6-SR	CCTAACACCTAATGTAATGGCGGCGG TTACTATTCCAATTGGTTC	GAACCAATTGGAATAGTAACCGCCGCCA TTACATTAGGTGTTAGG
pb6-270A	GAATTAACTGGTTCATTAACTGCTGC GGCGGCGGCGGCGTCTCTTGGTTCTA TGGAACTG	CAGTTCCATAGAACCAAGAGACGCCGCC GCCGCCGCAGCAGTTAATGAACCAGTTA ATTC
pb6-E	GATAAATCTCTTGGTTCTATGGCGCT GTATAAAGATCTTATCAAAAC	GTTTTGATAAGATCTTTATACAGCGCCAT AGAACCAAGAGATTTATC
pb6-K1	CTTGGTTCTATGGAACTGTATGCGGA TCTTATCAAAACTCTGAAG	CTTCAGAGTTTTGATAAGATCCGCATACA GTTCCATAGAACCAAG
pb6-K2	GGAACTGTATAAAGATCTTATCGCGA CTCTGAAGGTAGTTAACCG	CGGTTAACTACCTTCAGAGTCGCGATAAG ATCTTTATACAGTTCC
pb6-3K	GATAAATCTCTTGGTTCTATGGCGCT GTATGCGGATCTTATCGCGACTCTGG CGGTAGTTAACCGCTTTGAAATC	GATTTCAAAGCGGTTAACTACCGCCAGA GTCGCGATAAGATCCGCATACAGCGCCA TAGAACCAAGAGATTTATC
pb6-330A	GCGCACGTTAATATTCCTACTATTGC GGCGGCGGCGGCGGCGGCGACTTCA GTAGAATTTAAGGCTATTCCGTCAGC GGCGGCGGCTGGTGACGAAGGTTAC TTAG	CTAAGTAACCTTCGTCACCAGCCGCCGCC GCTGACGGAATAGCCTTAAATTCTACTGA AGTCGCCGCCGCCGCCGCCGCCGCAATA GTAGGAATATTAACGTGCGC

Mutants are colour coded as in Figure 4 and Supplementary Figure 1a.

Supplementary references

1. Schneider, C. A., Rasband, W. S. & Eliceiri, K. W. NIH Image to ImageJ: 25 years of image analysis. *Nat. Methods* **9,** 671–675 (2012).

2. Robert, X. & Gouet, P. Deciphering key features in protein structures with the new ENDscript server. *Nucleic Acids Res.* **42,** W320-324 (2014).

3. Holm, L. & Laakso, L. M. Dali server update. *Nucleic Acids Res.* **44,** W351-355 (2016).

4. Pell, L. G., Kanelis, V., Donaldson, L. W., Howell, P. L. & Davidson, A. R. The phage lambda major tail protein structure reveals a common evolution for long-tailed phages and the type VI bacterial secretion system. *Proc. Natl. Acad. Sci. U. S. A.* **106,** 4160–4165 (2009).

5. Kanamaru, S. *et al.* Structure of the cell-puncturing device of bacteriophage T4. *Nature* **415,** 553–557 (2002).
