# Supplementary material for: Bacteriophage T5 tail tube structure suggests a trigger mechanism for Siphoviridae DNA ejection
Source: Nat Commun. 2017 Dec 5;8:1953. doi: 10.1038/s41467-017-02049-3 (PMC5717097; doi:10.1038/s41467-017-02049-3)
Supplement: Supplementary file 3 — Description of Additional Supplementary Files [file 41467_2017_2049_MOESM3_ESM.pdf]

Description of Supplementary Files

File Name: Supplementary Movie 1
Description: **Comparison of the full and empty tail tubes.** EM reconstruction of T5 tail tube before (cyan) or after (magenta) interaction with T5 receptor at 6 Å resolution were superimposed in Chimera (correlation 0.98). The crystal structure of pb6 rigidly fitted into the density of the tube is shown as a yellow ribbon.

File Name: Supplementary Movie 2
Description: **Comparison of the empty tail tube and the tube fibres.** EM reconstruction of T5 tail tube after interaction with T5 receptor (magenta) and pb6 tube fibres (yellow) at 9 Å resolution were superimposed in Chimera (correlation 0.99). The crystal structure of pb6 rigidly fitted into the density of the tube is shown as a green ribbon.
